# An overview of human handedness in twins

**DOI:** 10.3389/fpsyg.2014.00010

**Published:** 2014-01-24

**Authors:** Syuichi Ooki

**Affiliations:** Department of Health Science, Ishikawa Prefectural Nursing UniversityKahoku, Japan

**Keywords:** twin study, laterality, handedness, brain asymmetry, zygosity, monozygotic twins, brain lateralization, human behavior

## Abstract

There has been a long-standing debate on the complex correlation between the development of human hand preference and brain lateralization. Handedness, used as a proxy for cerebral lateralization, is a topic of considerable importance because of its potential to reveal the mechanisms of the underlying pathophysiology of problems related to brain development or cognitive systems. Twin studies, which represent an important method of research in human genetics, would provide valuable suggestions to the studies on the relationship between lateralization and cognitive systems. Many studies have been performed using twin subjects; however, the results are inconsistent, partly because of sample size, background assumptions, data limits or inaccuracies, incorrect zygosity classification, and/or lack of birth histories. In summary, within the long history and large number of twin studies performed on handedness, a surprisingly large number of controversial findings have been reported, suggesting the complicated nature of this phenotype. In this mini review, the wide variety of twin studies on human handedness performed to date are introduced.

## INTRODUCTION

Approximately 90% of humans are right-handed, with the rest made up of left-handed and ambidextrous individuals. It is well established as to singletons that the prevalence of left-handedness in males is slightly higher than that in females (Papadatou-Pastou et al., 2008). There has been a long-standing debate on the complex correlation between the development of human hand preference and brain lateralization. Handedness, used as a proxy for cerebral lateralization, is a topic of considerable importance because of its potential to reveal the mechanisms of the underlying pathophysiology of problems related to brain development or cognitive systems. Twin studies, which represent an important method of research in human genetics, would provide valuable suggestions to the studies on the relationship between lateralization and cognitive systems.

This Mini Review summarizes the twin studies of human handedness laterality, and touches on the relevant research factors such as twin type, chorion and placentation types, zygosity, gender effects, birth-order effects, handedness discordance, and brain measures using magnetic resonance imaging (MRI) and functional MRI (fMRI). Genetic twin analyses of the origins of handedness were not included. The relationship between handedness and disease is omitted here, as it is beyond the scope of this brief review. Other human laterality, such as footedness, is also excluded because of an insufficient number of twin studies to date.

## TWINS AS SUBJECTS FOR THE STUDY OF HUMAN HANDEDNESS

There are two types of twins, and these types have completely different origins. Monozygotic (MZ) twins derive from the division of a single zygote, whereas dizygotic (DZ) twins derive from the independent release and subsequent fertilization of two ova (Machin, 1994). Handedness itself may be included as one of the anthropometric traits in similarity diagnosis (Segal, 1984).

It is commonly assumed that separation takes place in the early days of multicellular embryo development rather than at the initial zygote stage. If this split occurs within the first 72 h, the result is dichorionic monozygotic (DC-MZ) twin pregnancy. If the split takes place from 3 to 12 days after fertilization, a monochorionic monozygotic (MC-MZ) twin pregnancy is produced (Machin, 1994). Twin-twin transfusion syndrome (TTTS) is a serious condition that affects 10 to 15% of twin pregnancies with MC diamniotic placentation, resulting from the shunting of blood from one twin (the donor) to the other (the recipient) through placental vascular anastomoses.

MZ twin pairs share 100% of their DNA sequence, which means that most variation in pairs’ traits is due to their unique or shared environment. DZ twin pairs share about 50% of their polymorphisms. DZ twin pairs are helpful to study because they tend to share many aspects of their environment by virtue of being born in the same time and place. Twin studies help disentangle the relative importance of environmental and genetic influences on individual traits and behaviors by comparing the similarity of MZ and DZ twin pairs, although they may differ due to fetal development and birth histories. For example, large birth weight differences in TTTS may indicate neurological risk in the smaller twin.

## PREVALENCE BETWEEN TWINS AND SINGLETONS IN THE SAME POPULATION

Many studies (Tambs et al., 1987; Ellis et al., 1988; Williams et al., 1992; Coren, 1994; Davis and Annett, 1994) suggested that the prevalence of left-handedness is higher in twins compared to singletons for several hypothesized reasons, i.e., intrauterine crowding, mirror-imaging, and pre- and/or perinatal damage.

According to Springer and Searleman (1980), in a compilation of 15 studies of handedness distribution, the mean proportions of left-handedness are as follows: singletons, 8.5%; DZ twins, 14.0%; and MZ twins, 14.5%. However, twins and singletons are seldom: assessed using the same handedness criteria, recruited in the same manner, or matched for age and sex (McManus, 1980).

Twins are more likely than singletons to be born prematurely and/or to experience perinatal injuries, and it has been suggested that the increase in left-handedness in neurologically intact twins reflects one end of the spectrum of the pathological left-handedness syndrome as formulated by Satz (1972). They suggested that twin populations may include two types of left-handers: natural and pathological (although firm evidence to support this view is lacking, but cf. Gurd et al., 2013).

## SPECIFIC FACTORS FOR TWINS

### CHORION TYPE OR PLACENTATION

Ever since Newman (1928) it has been speculated that delayed embryo splitting of MZ is associated with mirror-imaging effects if the division occurs after the establishment of an axis of bilateral symmetry. In such a situation, opposite handedness in the same pair of twins is expected, and discordant pairs in terms of handedness are expected to be more frequent in MC-MZ than DC-MZ pairs.

In a sample of 44 pairs of MZ twin children, consisting of 23 MC-MZ and 21 DC-MZ selected from hospital records, 18% of MC-MZ pairs were discordant pairs, and 26% of DC-MZ pairs were discordant (Sokol et al., 1995). Carlier et al. (1996), using 20 MC-MZ and 24 DC-MZ twin pairs, also reported a similar tendency in that the MC-MZs and DC-MZs differed neither in the frequency of discordant pairs nor in handedness, laterality measurements, or manual performance, suggesting that there was no chorion type effect. The largest study to date by Derom et al. (1996), using 254 MC-MZ pairs and 121 DC-MZ pairs, found no chorion effect on left-handedness. Several twin studies use another useful proxy variable of chorion type, namely placentation. Medland et al. (2003) and Ooki (2006) both found no effects of placentation on handedness using large populations of twin subjects. However, handedness was not actually experimentally tested in these studies, which used only verbal report.

Reviews of the literature examining handedness in twins by McManus (1980) and Sicotte et al. (1999) found no support for the theory of mirror-imaging. It is possible that the early support of this theory may be confounded by the inaccuracy of zygosity determination, since some investigators regarded discordant handedness as a marker of zygosity (Carlier et al., 1996; Sicotte et al., 1999). Some 20–25% of MZ twin pairs have discordant handedness (McManus, 1980; McManus and Bryden, 1992; Annett, 2002). Although numerous individual cases of mirror-imaging twins with discordant handedness have been reported (Sommer et al., 1999, 2002), discordant handedness in MZ twin pairs is not currently thought to represent a mirror-imaging phenomenon in general.

### ZYGOSITY

If perinatal complication is related to left-handedness, the prevalence of left-handedness in MZ should be higher than that in DZ, because MZ, especially MC-MZ, biologically have more birth complications (Machin, 1994). With improvements in neonatal medicine, this may no longer be the case.

According to the review by McManus (1980) of 18 studies performed between 1924 and 1976, a total of 15% of 5,140 MZ and 13% of 4,436 DZ twins were left-handed. The only evidence in favor of MZ twins having a higher prevalence of left-handedness than DZ twins was obtained prior to 1930, when classification of laterality was not entirely independent of zygosity determination. It is clear that further study is warranted.

According to the meta-analysis of Sicotte et al. (1999), there exists no zygosity difference between MZ and DZ individuals, with a few individual outliers of the earlier studies. They concluded that there is nothing specific about the MZ twinning process *per se* that contributes to an excess of left-handedness in twins. Basso et al. (2000) found that there was a similar frequency of non-right-handedness in MZ (8.0%) and same-sex DZ (7.8%) twins born between 1900 and 1910. Orlebeke et al. (1996) observed a slightly higher prevalence of left-handedness in MZ male pairs (15%) compared to MZ female pairs (13%), DZ male pairs (13%), and DZ female pairs (13%). On the other hand, most recent studies with large populations (Medland et al., 2003; Ooki, 2006; Vuoksimaa et al., 2010) found no differences in handedness between MZ and DZ same-sex females nor between MZ and DZ same-sex males.

### SEX OF CO-TWIN: TESTOSTERONE HYPOTHESIS

According to Vuoksimaa et al. (2010), there exist two opposite hypotheses regarding testosterone levels and handedness. According to the Geschwind–Behan–Galaburda hypothesis (Geschwind and Behan, 1982; Geschwind and Galaburda, 1985), high levels of testosterone may inhibit the development of the left hemisphere and enhance the development of the right hemisphere. This can shift handedness and language functions from the left hemisphere to the right, resulting in weaker dextrality or left-handedness. Because testosterone is thought to pass between twins in utero, it was predicted that females with a male twin would show a high incidence of sinistrality compared to females with a female twin. Similarly, it was predicted that males with a male twin would be more likely to be sinistral than males with a female twin.

Götestam et al. (1992), in line with this hypothesis, reported that the prevalence of twins was lower among male homosexuals than in the general population, and explained that prenatal testosterone levels do not drop as dramatically in twins as they do in single fetuses, thereby counteracting the low levels of testosterone that could lead to homosexuality; a view not without controversy.

In contrast to the Geschwind–Behan–Galaburda hypothesis, an alternative theory suggests that left-handedness is caused by decreased levels of testosterone (Witelson and Nowakowski, 1991). This callosal theory proposes that low prenatal testosterone levels result in regressive development of the temporo-parietal regions of the brain, resulting in a larger isthmus of the corpus callosum and less functional asymmetry, thus increasing left-handedness. This view requires several leaps of faith: that testosterone is causally related to temporo-parietal development; that this in turn is causally related to callosal size, and that size of the callosal isthmus is causally related to functional asymmetry in the directions prescribed (cf. Gurd and Cowell, 2013; Gurd et al., 2013 for alternate lines of evidence, albeit on a small scale).

Three studies (Elkadi et al., 1999; Ooki, 2006; Vuoksimaa et al., 2010) have directly compared the rate of left-handedness between females from opposite-sex and same-sex twin pairs. According to Elkadi et al. (1999) measures of the strength of hand preference and the incidence of sinistrality revealed no difference between opposite-sex and same-sex twins for either sex. According to Ooki (2006), no effect of the sex of the co-twin was observed in either males or females. Medland et al. (2009) found no difference in the prevalence of left-handedness between twins from same-sex and opposite-sex pairs in a series of increasingly constrained models testing for differences in prevalence.

Vuoksimaa et al. (2010) tested for differences in the rates of left-handedness or right-handedness in female twins from same-sex and opposite-sex twin pairs. They found a significantly lower prevalence of left-handedness in females from opposite-sex pairs (5.3%) compared to females from same-sex pairs (8.6%). Their results support the callosal hypothesis and are difficult to fully explain by postnatal factors, but they offer support to the theory that relates testosterone to the formation of handedness, and in a population-based sample, are suggestive of the effects of prenatal testosterone transfer.

### BIRTH ORDER WITHIN TWINS: FIRST BORN vs. SECOND BORN

There are different risks to being first and to being second born within twin pairs – both carry risks. The first born has to prepare the birth canal, the second born, if larger and with a significant delay – may be at higher risk of anoxia – for vaginal deliveries. In a random sample of 104 pairs of handedness-discordant twins of 6 years of age or older, a significant relationship has been found between birth order and handedness in MZ twins, there being an excess of left-handed individuals among first-born twins. No such relation has been found in DZ twins (Christian et al., 1979). On the other hand, Boklage (1981) observed a 1.8-fold higher prevalence of left-handedness in the second-born members of same-sex discordant pairs, suggesting the secondary effect of hypoxia or acidosis. The effect of birth order within twin pairs has been intensively discussed (Orlebeke et al., 1996; James and Orlebeke, 2002). Most studies with large sample size (Derom et al., 1996; Elkadi et al., 1999; Medland et al., 2003; Ooki, 2006; Vuoksimaa et al., 2010) found no significant differences between first- and second-born twins in terms of left-handedness.

### HANDEDNESS DISCORDANT MZ TWIN STUDY: CO-TWIN CONTROL STUDY

MZ twin pairs with discordant handedness are as genotypically alike as it is possible to be, and it is therefore possible to study the consequences of phenotypical left or mixed handedness with the ideal set of controls: namely, the right-handed twin members.

Clark et al. (1986) attempted to determine if the discrepancy in measured intelligence between MZ twin pairs concordant for handedness differed measurably from the discrepancy between MZ twin pairs discordant for handedness. Eight sets of MZ twins were examined, and no evidence was found to support the influence of pathogenic congenital factors on handedness. Segal (1989) compared three types of IQ scores for 67 young MZ twin pairs organized according to concordance or discordance for handedness and relative birth weight. The results support the hypothesis that left-handedness in lower-birth-weight MZ co-twins may be associated with pre-natal pathological events, while left-handedness in higher-birth-weight left-handed MZ co-twins may be associated with delayed zygotic splitting and disrupted asymmetry determination. Jäncke and Steinmetz (1995) examined 20 MZ twin pairs of whom 10 pairs were concordantly right-handed and 10 pairs discordant for handedness to determine whether the absolute degree of asymmetry of hand motor performance may have a heritable component. They found that at least in MZ twins the degree of hand motor asymmetry is mainly determined by non-genetic factors, whereas overall hand motor skill is more likely to be influenced by genetic factors. Kee et al. (1998) constituted a multitask appraisal of cerebral hemisphere specialization with 13 MZ twin pairs discordant for handedness, and found that asymmetries for left- and right-handed MZ twins were more similar to patterns reported in the literature for left- and right-handed singletons, respectively, than for opposite-handed co-twins. Gurd et al. (2006) examined 20 female MZ twin pairs discordant for handedness, and found that in the hand-preference inventories, the right-handers were more strongly lateralized that their left-handed sisters, and that the left-handers had greater variation in their laterality scores. They concluded that the analyses not only revealed obvious strong main effects of writing hand on performance tasks, but also interaction effects of handedness on the peg-moving task. Gurd et al. (2013) recently, using 26 handedness discordant MZ twin pairs, reported significant correlation between language-specific functional laterality in inferior and middle frontal gyri, and anterior corpus callosum. Häberling et al. (2012), using 35 MZ twin pairs of whom 17 pairs were concordant for handedness and 18 pairs discordant for handedness, suggested that handedness and hemispheric dominance for speech production might be at least partly dependent on genetically controlled processes of axonal pruning in the corpus callosum.

### HANDEDNESS AND OTHER ASYMMETRY MEASURED BY MRI/fMRI

Several researchers used handedness as a marker of intrauterine neurological development and compared the handedness and brain asymmetry using fMRI. MZ twin pairs are often examined as an initial step toward documenting the nature of laterality. The report of Sommer et al. (1999) pointed out that in diseases in which cerebral lateralization is important to the pathology, the assumption that MZ twins share cerebral hemispherical functions is false due to the occurrence of mirror-imaging.

Sommer et al. (2002) studied language lateralization measured by fMRI in 12 MZ twin pairs who were concordant for handedness and 13 MZ twin pairs discordant for handedness, and claimed that high intra-pair correlation for language lateralization in the handedness-concordant twins suggests a genetic basis for language lateralization, although they did not indicate the ages of the twins tested. However, in MZ twin pairs with discordant handedness, discordance for language lateralization occurs in a significant number of twins, consistent with a view that discordant language dominance may be caused by a relatively late splitting of the original embryo. Lux et al. (2008) examined the nature of hemispheric lateralization for neural processes underlying verbal fluency and visuo-spatial attention using a single pair of handedness discordant MZ twins. They found that the right-handed twin had left-lateralized verbal with right-lateralized visuo-spatial attention, while the left-handed twin had right-lateralized verbal with left-lateralized visuo-spatial activation. Rosch et al. (2010) examined cerebellar asymmetry in a pair of MZ handedness-discordant twins and found that the left-handed twin showed clockwise directional torque in the cerebral and cerebellar regions, while the right-handed twin showed disparate directions of cerebral (counter-clockwise) vs. cerebellar (clockwise) torque.

Geschwind et al. (2002) measured frontal, temporal, parietal, and occipital brain volumes and examined the relationship between cerebral asymmetry and handedness of 72 MZ and 67 DZ twin pairs. They found that genetic factors contributed twice the influence to left and light cerebral hemispheric volumes in the non-right-handed twin pairs.

## CONCLUSION

It is often said that twin study represents a way of doing experimental research in a natural setting. Many studies have been performed using twin subjects; however, the results are inconsistent, partly because of the small sample size used or faulty assumptions in theoretical models (cf. McManus et al., 2013). Nevertheless, almost all studies that have examined the prevalence of twins in the general population have shown a higher frequency of left-handedness in twins than in singletons (Vuoksimaa et al., 2009). With recent advances in neuroimaging, such as MRI, fMRI, and DTI (diffusion tensor imaging), many co-twin control studies on the relationship between handedness and brain asymmetries have been intensively performed (Badzakova-Trajkov et al., 2010; Häberling et al., 2013; Gurd et al., 2013; Gurd and Cowell, 2013). But the sample size is still not very large and hence the statistical power is insufficient and some important information related to cerebral asymmetry or handedness is not always presented. Considering the small sample size, not only a heritability study, but also more detailed case reports, especially including the pre-/perinatal conditions of each twin, may be useful. In a recent review of cerebral asymmetry and language development, Bishop (2013) argued that before we can grasp the opportunities presented by technological developments in neuroscience and genetics, we need to do basic research to clarify how best to conceptualize and reliably measure cerebral asymmetry.

While twin study may not provide conclusive evidence as to the origins or mechanisms between human handedness and brain development or cognitive process, it indubitably provides an important first step to clarify these problems. It is essential however that neurodevelopmental factors specific to twinnedness be included in the analysis (cf. Gurd et al., 2013; Gurd and Cowell, 2013).

## Conflict of Interest Statement

The author declares that the research was conducted in the absence of any commercial or financial relationships that could be construed as a potential conflict of interest.
